# Fabrication of 3D Ordered Structures with Multiple Materials via Macroscopic Supramolecular Assembly

**DOI:** 10.1002/advs.202002025

**Published:** 2020-10-16

**Authors:** Qian Zhang, Yingzhi Sun, Chengzhi He, Feng Shi, Mengjiao Cheng

**Affiliations:** ^1^ State Key Laboratory of Chemical Resource Engineering & Beijing Laboratory of Biomedical Materials & Beijing Advanced Innovation Center for Soft Matter Science and Engineering Beijing University of Chemical Technology Beijing 100029 China

**Keywords:** additive manufacture, flexible spacing coating, heterogeneous materials, macroscopic supramolecular assembly, multivalent host/guest interactions

## Abstract

Integration of diverse materials into 3D ordered structures is urgently required for advanced manufacture owing to increase in demand for high‐performance products. Most additive manufacturing techniques mainly focus on simply combining different equipment, while interfacial binding of distinctive materials remains a fundamental problem. Increasing studies on macroscopic supramolecular assembly (MSA) have revealed efficient interfacial interactions based on multivalency of supramolecular interactions facilitated by a “flexible spacing coating.” To demonstrate facile fabrication of 3D heterogeneous ordered structures, the combination of MSA and magnetic field‐assisted alignment has been developed as a new methodology for in situ integration of a wide range of materials, including elastomer, resin, plastics, metal, and quartz glass, with modulus ranging from tens of MPa to over 70 GPa. Assembly of single material, coassembly of two to four distinctive materials, and 3D alignment of “bridge‐like” and “cross‐stacked” heterogeneous structures are demonstrated. This methodology has provided a new solution to mild and efficient assembly of multiple materials at the macroscopic scale, which holds promise for advanced fabrication in fields of tissue engineering, electronic devices, and actuators.

## Graphical abstract

Integration of diverse materials into 3D ordered structures is urgently required in advanced manufacture to realize multiple functions in high‐performance products. A new principle is developed based on macroscopic supramolecular assembly (MSA) by combining efficient interfacial interactions with magnetic field‐assisted alignment. Assembly of distinctive rigid materials and 3D alignment of “bridge‐like” or “cross‐stacked” heterogeneous structures are demonstrated.

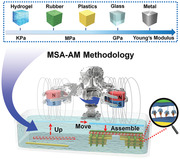

Additive manufacture, especially three dimentional (3D) printing, is a revolutionary concept with applications in diverse fields.^[^ ^1^, ^2^, ^3^, ^4^ ^]^ Owing to the increase in demand for differently processed materials with multiple functions for advanced applications such as tissue engineering, the research of additive manufacture is facing new challenges of integrating multicomponents into one entity with high‐performance or desired functions.^[^ ^5^, ^6^ ^]^ For example, ex situ cell growth and tissue formation require complex 3D microenvironments consisting of multiple materials with varied mechanical properties, alignment, and surface chemistry; soft electronics or actuators normally require combination of almost incompatible materials with distinctive processing conditions, such as metal and polymer.^[^ ^7^, ^8^, ^9^ ^]^ Currently, two general strategies are used for processing multiple materials: 1) designing devices with multiple nozzles in extrusion^[^ ^10^, ^11^ ^]^/jetting^[^ ^12^ ^]^ additive manufacture processes, which is mainly limited to cross‐linkable or curable fluidic systems, and 2) applying a “multiprocess 3D printing” method by incorporating several equipment of additive manufacture into a production line, where multiple materials are actually physically combined.^[^ ^13^, ^14^ ^]^ Although these strategies can lead to the formation of heterogeneous structures to some extent, designing of in situ integrated distinctive materials with totally different physical/chemical properties and processing conditions (e.g., metal, plastic, and hydrogel) is still challenging.^[^ ^15^ ^]^ Therefore, developing a new principle or methodology for fabricating heterogeneous ordered structures of multiple materials is urgently required to overcome the limitations of current additive manufacture methods and enrich the diversity of 3D structures, which may broaden the frontier applications of additive manufacture in tissue engineering and soft electronics.

Macroscopic supramolecular assembly (MSA), a result of the recent progress in supramolecular science, can assist in integrating multiple materials into ordered structures.^[^ ^16^, ^17^, ^18^, ^19^ ^]^ MSA refers to the study of multivalent interactions between building blocks of size exceeding 10 µm and modified with supramolecular groups,^[^ ^20^, ^21^, ^22^, ^23^, ^24^ ^]^ which is an ideal platform for modular assembly and a potentially new principle of additive manufacture for preparing designated structures for the following reasons: 1) building blocks with size larger than 10 µm can be tailored into required sizes, shapes, and surface chemistry owing to the feasibility of mature micro‐/nanofabrication methods; 2) these building blocks can be subsequently manipulated via noncontact methods, as MSA is highly compatible with various micromanipulation methods such as those involving magnetic/electric field; 3) finally, the interfacial supramolecular interactions between macroscopic building blocks ensure a robust structure, allowing for further stepwise assembly. As most current MSA reports are mainly limited to soft materials, typically gel systems,^[^ ^25^, ^26^ ^]^ the above principle of additive manufacture involving MSA relies on the breakthrough discovery of a versatile MSA mechanism for assembling a wide range of materials, especially those with distinctive properties. The key to realize MSA of incompatible rigid surfaces is to improve the compliance of both the interactive groups and heterogeneous surfaces. Therefore, in this study, we have proposed a rapid assembly strategy of distinctive materials based on a compliant “flexible spacing coating” method^[^ ^27^, ^28^ ^]^ and developed a new methodology of additive manufacture based on MSA (MSA‐AM) to construct heterogeneous assemblies and 3D ordered structures.

By premodifying a flexible spacing coating beneath supramolecular groups, we achieved the MSA of heterogeneous materials with high modulus, ranging from tens of MPa to over 70 GPa, including elastomer, plastics, metal, and quartz glass. Based on the versatility of MSA of multiple materials, we have further demonstrated the merits of the MSA‐AM methodology by combining magnetic field‐assisted alignment and MSA for 3D manipulation and stabilization of desired building blocks, leading to heterogeneous structures consisting of some frequently used materials in tissue scaffolds. We envision that this MSA‐AM methodology will establish a novel principle of additive manufacture that will address the problems associated with processing of multiple materials and advanced fabrication of complex tissue scaffolds.

The first step toward fabricating 3D heterogeneous structures is to demonstrate the applicability of MSA to a wide range of materials. Therefore, we started with MSA of diverse materials such as elastomer, plastics, metal, and inorganic materials, namely, polyurethane (PU), polyethylene (PE), polypropylene (PP), polystyrene (PS), acrylonitrile butadiene styrene (ABS), aluminum (Al), and quartz glass. These materials were machined into millimeter‐scaled cubic shapes as building blocks of MSA. Then, we applied a layer‐by‐layer (LbL) technique to modify the supramolecular groups of *β*‐cyclodextrin (CD) or azobenzene (Azo) onto these building blocks via repeated cycles of alternate immersion in aqueous solutions of polycation/polyanion. In particular, we LbL‐assembled poly(diallyldimethylammonium chloride) (PDDA) and poly(acrylic acid) (PAA) grafted with CD or Azo (noted as PAA‐CD or PAA‐Azo) (**Figure** **1**a) onto building blocks until the designated number of multilayers was reached, indicated as (PDDA/PAA‐CD)_5_ and (PDDA/PAA‐Azo)_5,_ which represented the host and guest building blocks, respectively. Subsequently, we investigated the MSA behaviors of a material (e.g. ABS) by placing a pair of host/guest building blocks away from each other at the bottom of a beaker containing 15 mL water, followed by shaking on a rotating shaker at 180 rpm for 5 min (Scheme S1, Supporting Information). However, no assembly was observed for any material, including ABS (Figure 1b), PU, PE, PP, PS, Al, and quartz glass (Figure S1, Supporting Information), i.e., the building blocks were always separate even after prolonged shaking. These results highlighted a known disadvantage of MSA, that of being limited to soft gel building blocks, while rigid surfaces simply modified with interactive supramolecular groups did not undergo MSA. We believe that the assembly failure was due to the weak interfacial binding between macroscopic rigid surfaces for the following reasons: 1) the molecular motility of the supramolecular groups on the rigid surfaces is limited, hindering them from reaching molecular interactive distances;^[^ ^27^ ^]^ 2) the applied materials have considerably higher modulus (Figure 1d) than that of hydrogel systems, leading to poor surface compliance for self‐adaption in interfacial binding events.^[^ ^25^ ^]^ Taken together, MSA of these rigid materials is a fundamental problem that should be addressed with priority.

**Figure 1:**
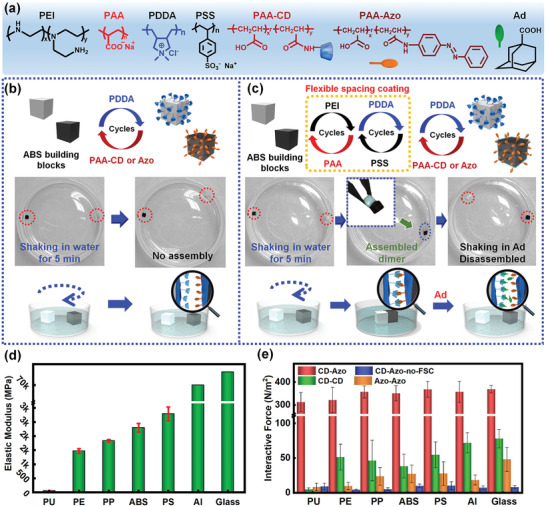
a) Schematic illustration of all used molecules for surface modification and disassembly. MSA experiments of acrylonitrile butadiene styrene (ABS) building blocks b) when only modified with supramolecular multilayers of (PDDA/PAA‐CD)_5_ or (PDDA/PAA‐Azo)_5_ and c) when introduced with a flexible spacing coating (FSC) beneath the supramolecular multilayers. d) Elastic moduli of different building blocks. e) Interactive forces versus varied materials when modified with (noted as CD‐Azo, CD‐CD and Azo‐Azo) and without FSC (noted as CD‐Azo‐no‐FSC).

Considering that the MSA efficiency mainly relies on a compliant surface and fast interfacial multivalency, we applied a flexible spacing coating that exhibits a highly flowing property beneath the supramolecular groups, and anticipated an improved surface compliance and molecular motility. In particular, we premodified a thick polyelectrolyte multilayer of poly(ethyleneimine) (PEI)/PAA, which is reported to show high flowability^[^ ^29^, ^30^, ^31^ ^]^ with increased LbL cycles but also accompanied with its increased stickiness; therefore, we further applied a barrier multilayer of PDDA/poly(sodium‐*p*‐styrenesulfonate) (PSS) to isolate nonselective stickiness of the PEI/PAA multilayer;^[^ ^32^ ^]^ the resulted composite multilayer of PEI/PAA‐PDDA/PSS is used as the flexible spacing coating, which has a low glass transition temperature and a low Young's modulus to provide a compliant surface with high molecular motility (Figure S2, Supporting Information). Subsequently, we further modified (PDDA/PAA‐CD)_5_ or (PDDA/PAA‐Azo)_5_ multilayers as the outermost supramolecular layer (Figure 1c) by using the feasibility of grafting supramolecular groups onto PAA. Following a similar MSA procedure of shaking a pair of host/guest building blocks in water, we observed rapid formation of a dimer assembled from the two building blocks in the presence of the flexible spacing coating; the dimer was robust enough to be lifted out of the water immediately after assembly and was intact even when shaken in air. Control MSA experiments of building blocks with and without the flexible spacing coating of the PEI/PAA‐PDDA/PSS multilayer have confirmed the dominant effect of the flexible spacing coating on MSA of diverse rigid materials. Thus, using MSA facilitated by a flexible spacing coating, we have demonstrated the applicability of MSA to a wide range of materials, including ABS, PU, PE, PP, PS, Al, and quartz glass (Figure S3, Supporting Information).

Although rigid materials undergo MSA, the mechanism underlying the assembly remains unclear, especially the contribution of supramolecular interactions to MSA. Therefore, we have conducted control experiments where 1) MSA occurred between identical host or guest building blocks and 2) disassembly of host/guest dimers was induced via free competitive guest molecules, such as 1‐adamantanecarboxylic acid (Ad). The results showed that two identical building blocks of the same surface chemistry did not lead to any assembly (Figure S4, Supporting Information), thereby excluding the possibility of nonselective adhesion and confirming the necessity of supramolecular interactions in MSA. Furthermore, the former CD/Azo dimer was totally disassembled when transferred to an Ad solution (Figure 1c) because of the competitive molecular interaction of CD/Ad (1500 M^−1^)^[^ ^17^ ^]^ over CD/Azo (770 M^−1^);^[^ ^33^ ^]^ disassembly could not have occurred if the interface assembly was due to non‐selective adhesion caused by polymer entanglement. Taken together, the supramolecular interaction is important for triggering MSA of rigid building blocks.

To quantify the contributions of both the flexible spacing coating and supramolecular interactions to MSA, we applied an in situ method of force measurement (Figure S5, Supporting Information), which determined the interfacial binding strength between rigid materials of varied surface modification by recording in real‐time the force change during the approach‐separation process of the two building blocks. For each material, we compared force values of four independent interactive pairs, including control groups without flexible spacing coatings (marked as CD‐Azo‐no‐FSC) and with flexible spacing coatings (marked as CD‐Azo, CD‐CD, and Azo‐Azo) (Figure 1e). The interactive force of CD‐Azo with flexible spacing coatings was remarkably larger than that of CD‐Azo‐no‐FSC, CD‐CD, and Azo‐Azo, indicating that absence of either the flexible spacing coating or supramolecular interactions weakened the binding strength and led to MSA failure. Moreover, the LbL deposition cycle number of the PEI/PAA multilayer shows a positive correlation with the interactive force (Figure S6, Supporting Information) due to its improved compliance and flowability favorable for facilitating interfacial binding. Taken together, both qualitative MSA behaviors and the corresponding quantitative results confirmed the dominant effects of the flexible spacing coating and supramolecular interactions on efficient interfacial binding between rigid materials.

As MSA can be applied to rigid materials, we speculated whether coassembly of different materials was possible, which would be important for the fabrication of heterogeneous structures. In particular, the compatibility of distinctive surfaces and the stability of assembled interfaces should be considered when utilizing diverse surface properties such as different surface compliance, wettability, and molecular motility. To determine the feasibility of MSA of surfaces made of different materials, we investigated coassembly between materials with either similar or opposite intrinsic wettability such as assembly pairs of PU‐ABS (hydrophobic/hydrophobic), Al‐Glass (hydrophilic/hydrophilic), and PU‐Glass or PU‐Al (hydrophobic/hydrophilic). Following the developed surface modification procedure of the flexible spacing coating and supramolecular groups, we obtained host/guest building blocks of the above combinations of materials. To assess the coassembly behavior, we placed a host building block of Al and a guest building block of quartz glass away from each other within water (**Figure** **2**a). After shaking at 180 rpm for about 5 min, the Al and quartz glass assembled into an intact dimer, indicating that MSA facilitated by the flexible spacing coating can be used for assembly of heterogeneous materials. One explanation for this phenomenon is that the introduction of the flexible spacing coating and supramolecular layers has weakened surface effects of intrinsic materials, which is favorable for efficient binding of heterogeneous surfaces via interfacial multivalency. To verify the versatility of this coassembly, we have further assessed other combinations of PU‐ABS, PU‐Glass, and PU‐Al, and obtained assemblies with similar efficiency (Figure 2b–d), indicating that the MSA methodology is versatile and applicable to diverse materials with a wide modulus range. As expected, we obtained a “trimer” of PU‐Al‐Glass (Figure 2e) and a linear “tetramer” of “Al‐Glass‐PU‐ABS” (Figure 2f; Figure S7, Supporting Information) following a similar protocol, which will be important for integrating multiple materials into one entity. MSA of larger heterogeneous structures at centimeter scale is also applicable (Figure S8, Supporting Information). Moreover, the binding strength of these assembled structures of heterogeneous materials has been measured via the in situ force measurement and summarized as Figure S5d (Supporting Information).

**Figure 2:**
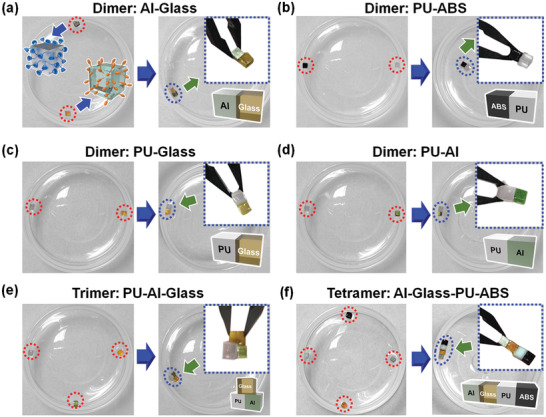
Dimers, trimer, and tetramer coassembled through distinctive materials. Building blocks modified with the flexible spacing coating (FSC) beneath a (PDDA/PAA‐CD)_5_ multilayer is noted as a host; meanwhile building blocks with the FSC beneath a (PDDA/PAA‐Azo)_5_ multilayer is noted as a guest. “Host–guest” dimers of a) Al‐Glass, b) PU‐ABS, c) PU‐Glass, and d) PU‐Al. e) A “host–guest–guest” trimer of PU‐Al‐Glass (PU has the outmost CD groups; Al and glass have the outmost Azo groups). f) A “host–guest–host–guest” tetramer of Al‐Glass‐PU‐ABS (Al and PU have CD at outmost; PU and ABS have Azo at outmost). Surface chemistry of the above building blocks is detailed in Table S1 (Supporting Information).

To demonstrate the fabrication of 3D ordered heterogeneous structures using MSA, we have developed a magnetic field‐assisted MSA methodology based on a new principle of additive manufacture (MSA‐AM) for both precise alignment and strong interfacial binding of designated building blocks. As a heterogeneous scaffold structure with controlled spatial alignment of different materials is highly desired in tissue engineering research, we attempted to assemble three materials typically used in fabrication of tissue scaffolds, namely, polydimethylsiloxane (PDMS), poly(ethylene terephthalate) (PET), and titanium (Ti), which are often used as tissue supports of artificial tendon,^[^ ^34^ ^]^ ligament^[^ ^35^ ^]^, and bone,^[^ ^36^ ^]^ respectively (**Figure** **3**a). To mimic the microenvironment within a comparable length scale of cells, we have reduced the size of the building blocks to hundreds of micrometers and processed them into strip‐like geometry (Figure S9, Supporting Information); to develop magnet‐responsive building blocks, we incorporated magnetic nanoparticles in the building blocks via either pre‐embedding or LbL surface modification (Figures S10 and S11, Supporting Information). Finally, the building blocks were modified with the flexible spacing coating and supramolecular groups using the LbL procedures as described earlier, leading to strip building blocks of PDMS, PET, and Ti decorated with either CD or Azo groups.

**Figure 3:**
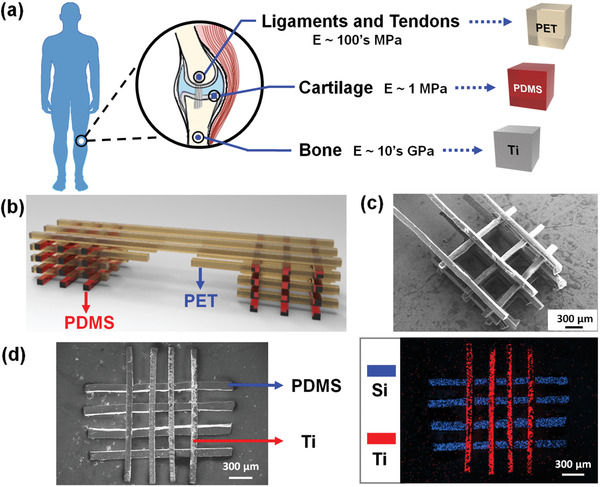
Schematic illustration of a) rigid materials used in tissue engineering and b) an example of the design of spatial alignment of distinctive materials. SEM images of heterogeneous 3D structures with c) bridge‐like and d) cross‐stacked spatial geometry. The corresponding energy dispersive spectrometer of element map displays the heterogeneous feature of the cross‐stacked structure.

The stepwise magnetic field‐assisted MSA‐AM methodology is schematically illustrated in **Scheme** **1**: 1) a Ti building block was dispersed in deionized water and further magnetically manipulated into designated locations; 2) with the draining of water, the Ti and substrate surfaces reached molecular interactive distance to trigger MSA, which stabilized the building blocks via improved interfacial binding due to the flexible spacing coating and molecular interactions; 3) more building blocks followed alternate processes of magnetic manipulation and MSA to form a designated structure; 4) during the fabrication process, we mildly and facilely tailored the material category, alignment, and spatial position of each building block within the structure, allowing flexible designing of diverse 3D heterogeneous structures. For example, the soft‐rigid combination of PDMS‐PET could be assembled into a “bridge‐like” structure (Figure 3b,c). The alternately well‐aligned soft/rigid layers of PDMS/PET building blocks ensured both strong support and energy dissipation, which rendered the “bridge” structure intact even with support‐free suspending across a long distance. Furthermore, we successfully fabricated an ordered structure with two distinctive materials, polymer (PDMS) and metal (Ti); the PDMS and Ti building blocks were orthogonally cross‐stacked through different layers, the strip‐like building blocks in each of which were aligned parallelly (Figure 3d). The overlaid elemental mapping image showed clear distribution of Si and Ti in the horizontally and vertically aligned PDMS and Ti building blocks. We have measured the location precision with a center‐to‐center distance deviation at around 3.33 µm and an angular deviation of 0.87° (Figure S12, Supporting Information). The combination of Ti and PDMS with spatial control over surface modulus holds promise to create a 3D microenvironment with matching high and low stiffness for osteoblasts^[^ ^37^ ^]^ and chondrocytes,^[^ ^38^ ^]^ respectively, thereby increasing its potential for application in tissue engineering in future. The complete fabrication process of a 3D ordered heterogeneous structure (PDMS and PET) via this MSA‐AM method is demonstrated in Video S1 (Supporting Information). But the current MSA‐AM methodology remains to be improved on the assembly efficiency and “intelligence” to realize continuous additive manufacture. For example, fast programmable assembly should be possible by combining MSA‐AM with smart recognition of targeted building blocks and automatic systems of robotic manipulation.

**Scheme 1:**
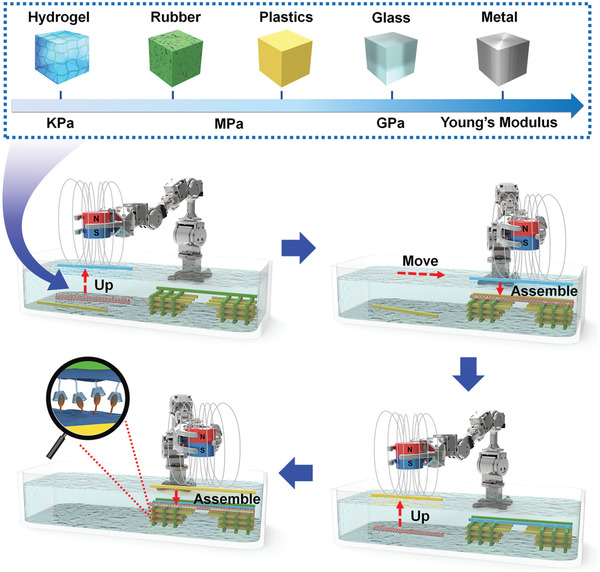
Illustration of the MSA‐AM methodology for in situ integration of multiple materials into 3D ordered structures.

In summary, we have developed an MSA‐AM methodology for integrating multiple materials into ordered structures by combining MSA of rigid materials, via the flexible spacing coating, with magnetic field‐assisted alignment. The presence of the flexible spacing coating remarkably improved the interfacial interactions of diverse materials, including PU, ABS, Al, metal, and quartz glass, leading to efficient MSA and high binding strength; furthermore, coassembly of two to four distinctive materials were demonstrated, irrespective of their different intrinsic surface properties. In addition, we have fabricated heterogeneous structures of “bridge‐like” and “cross‐stacked” geometry with 3D ordering and fine spatial control using magnetic field manipulation of building blocks. We envision that the unique features of the MSA‐AM methodology, which enabled in situ integration of multiple materials into well‐defined 3D structures, will advance the field of additive manufacture; furthermore, a flexible and mild control over material category, spatial alignment, and distribution may assist in designing and fabricating complex 3D heterogeneous structures for tissue engineering, electronic devices, and actuators.

## Conflict of Interest

The authors declare no conflict of interest.

## Supporting information

Supporting Information

Supplemental Video 1

## References

[1] A. J. Capel , R. P. Rimington , M. P. Lewis , S. D. R. Christie , Nat. Rev. Chem. 2018, 2, 422.

[2] A. Ambrosi , M. Pumera , Chem. Soc. Rev. 2016, 45, 2740. 27048921 10.1039/c5cs00714c

[3] R. L. Truby , J. A. Lewis , Nature 2016, 540, 371. 27974748 10.1038/nature21003

[4] K. Fu , Y. Yao , J. Dai , L. Hu , Adv. Mater. 2017, 29, 1603486. 10.1002/adma.201603486 27982475

[5] M. Rafiee , R. D. Farahani , D. Therriault , Adv. Sci. 2020, 7, 1902307. 10.1002/advs.201902307 PMC7312457 32596102

[6] A. Velasco‐Hogan , J. Xu , M. A. Meyers , Adv. Mater. 2018, 30, 1800940. 10.1002/adma.201800940 30133816

[7] M. Wehner , R. L. Truby , D. J. Fitzgerald , B. Mosadegh , G. M. Whitesides , J. A. Lewis , R. J. Wood , Nature 2016, 536, 451. 27558065 10.1038/nature19100

[8] Y.‐S. Kim , M. Mahmood , Y. Lee , N. K. Kim , S. Kwon , R. Herbert , D. Kim , H. C. Cho , W.‐H. Yeo , Adv. Sci. 2019, 6, 1900939. 10.1002/advs.201900939 PMC6724359 31508289

[9] X. Kuang , D. J. Roach , J. Wu , C. M. Hamel , Z. Ding , T. Wang , M. L. Dunn , H. J. Qi , Adv. Funct. Mater. 2019, 29, 1805290.

[10] X. Liu , H. Yuk , S. Lin , G. A. Parada , T.‐C. Tang , E. Tham , C. de la Fuente‐Nunez , T. K. Lu , X. Zhao , Adv. Mater. 2018, 30, 1704821. 10.1002/adma.201704821 29205532

[11] M. A. Skylar‐Scott , J. Mueller , C. W. Visser , J. A. Lewis , Nature 2019, 575, 330. 31723289 10.1038/s41586-019-1736-8

[12] B. W. An , K. Kim , H. Lee , S.‐Y. Kim , Y. Shim , D.‐Y. Lee , J. Y. Song , J.‐U. Park , Adv. Mater. 2015, 27, 4322. 26095718 10.1002/adma.201502092

[13] M. S. Hossain , J. A. Gonzalez , R. M. Hernandez , M. A. I. Shuvo , J. Mireles , A. Choudhuri , Y. Lin , R. B. Wicker , Addit. Manuf. 2016, 10, 58.

[14] A. M. Gaikwad , G. L. Whiting , D. A. Steingart , A. C. Arias , Adv. Mater. 2011, 23, 3251. 21661062 10.1002/adma.201100894

[15] E. MacDonald , R. Wicker , Science 2016, 353, aaf2093. 27708075 10.1126/science.aaf2093

[16] M.‐J. Cheng , Q. Zhang , F. Shi , Chin. J. Polym. Sci. 2018, 36, 306.

[17] A. Harada , R. Kobayashi , Y. Takashima , A. Hashidzume , H. Yamaguchi , Nat. Chem. 2011, 3, 34. 21160514 10.1038/nchem.893

[18] M. Cheng , H. Gao , Y. Zhang , W. Tremel , J.‐F. Chen , F. Shi , W. Knoll , Langmuir 2011, 27, 6559. 21542598 10.1021/la201399w

[19] M. Cheng , F. Shi , Acta Polym. Sin. 2020, 51, 598.

[20] C. Ma , T. Li , Q. Zhao , X. Yang , J. Wu , Y. Luo , T. Xie , Adv. Mater. 2014, 26, 5665. 24975743 10.1002/adma.201402026

[21] G. Ju , F. Guo , Q. Zhang , A. J. C. Kuehne , S. Cui , M. Cheng , F. Shi , Adv. Mater. 2017, 29, 1702444. 10.1002/adma.201702444 28782850

[22] J. Li , Z. Xu , Y. Xiao , G. Gao , J. Chen , J. Yin , J. Fu , J. Mater. Chem. B 2018, 6, 257. 32254168 10.1039/c7tb02904g

[23] H. Qi , M. Ghodousi , Y. Du , C. Grun , H. Bae , P. Yin , A. Khademhosseini , Nat. Commun. 2013, 4, 2275. 24013352 10.1038/ncomms3275 PMC3768014

[24] Y. Han , Y. Yang , S. Li , J. Wu , Y. Chen , T. J. Lu , F. Xu , Biofabrication 2013, 5, 035004. 23715009 10.1088/1758-5082/5/3/035004

[25] G. Ju , M. Cheng , F. Guo , Q. Zhang , F. Shi , Angew. Chem., Int. Ed. 2018, 57, 8963. 10.1002/anie.201803632 29851216

[26] G. Ju , M. Cheng , Q. Zhang , F. Guo , P. Xie , F. Shi , ACS Appl. Nano Mater. 2018, 1, 5662.

[27] M. Cheng , F. Shi , J. Li , Z. Lin , C. Jiang , M. Xiao , L. Zhang , W. Yang , T. Nishi , Adv. Mater. 2014, 26, 3009. 24453055 10.1002/adma.201305177

[28] Q. Zhang , C. Liu , G. Ju , M. Cheng , F. Shi , Macromol. Rapid Commun. 2018, 39, 1800180. 10.1002/marc.201800180 29749034

[29] X. Wang , F. Liu , X. Zheng , J. Sun , Angew. Chem., Int. Ed. 2011, 50, 11378. 10.1002/anie.201105822 22113797

[30] P. J. Yoo , K. T. Nam , J. Qi , S.‐K. Lee , J. Park , A. M. Belcher , P. T. Hammond , Nat. Mater. 2006, 5, 234. 16489350 10.1038/nmat1596

[31] P. J. Yoo , N. S. Zacharia , J. Doh , K. T. Nam , A. M. Belcher , P. T. Hammond , ACS Nano 2008, 2, 561. 19206583 10.1021/nn700404y

[32] J. M. Garza , P. Schaaf , S. Muller , V. Ball , J.‐F. Stoltz , J.‐C. Voegel , P. Lavalle , Langmuir 2004, 20, 7298. 15301518 10.1021/la049106o

[33] H. Yamaguchi , Y. Kobayashi , R. Kobayashi , Y. Takashima , A. Hashidzume , A. Harada , Nat. Commun. 2012, 3, 603. 22215078 10.1038/ncomms1617 PMC3272571

[34] Q. Li , L. Sun , L. Zhang , Z. Xu , Y. Kang , P. Xue , J. Biomed. Mater. Res., Part A 2018, 106, 408. 10.1002/jbm.a.36254 28971550

[35] Y. Zhi , J. Jiang , P. Zhang , S. Chen , Artif. Organs 2019, 43, E94. 30412273 10.1111/aor.13389

[36] M. Zhai , Y. Zhu , M. Yang , C. Mao , Adv. Sci. 2020, 2001334. 10.1002/advs.202001334 PMC7539212 33042751

[37] Z. Schwartz , R. Olivares‐Navarrete , M. Wieland , D. L. Cochran , B. D. Boyan , Biomaterials 2009, 30, 3390. 19395022 10.1016/j.biomaterials.2009.03.047 PMC2700751

[38] T. Zhang , T. Gong , J. Xie , S. Lin , Y. Liu , T. Zhou , Y. Lin , ACS Appl. Mater. Interfaces 2016, 8, 22884. 27534990 10.1021/acsami.6b07097

