# Supplementary material for: Fabrication of 3D Ordered Structures with Multiple Materials via Macroscopic Supramolecular Assembly
Source: Adv Sci (Weinh). 2020 Oct 16;7(23):2002025. doi: 10.1002/advs.202002025 (PMC7709987; doi:10.1002/advs.202002025)
Supplement: Supplementary file 1 — Supporting Information [file ADVS-7-2002025-s001.pdf]

© 2020 Wiley-VCH GmbH

ADVANCED SCIENCE
Open Access

Supporting Information

for *Adv. Sci.,* DOI: 10.1002/advs.202002025

Fabrication of 3D Ordered Structures with Multiple Materials via Macroscopic Supramolecular Assembly

*Qian Zhang, Yingzhi Sun, Chengzhi He, Feng Shi, and Mengjiao Cheng**

Supporting Information

Fabrication of 3D Ordered Structures with Multiple Materials Through Macroscopic Supramolecular Assembly

*Qian Zhang, Yingzhi Sun, Chengzhi He, Feng Shi and Mengjiao Cheng**

State Key Laboratory of Chemical Resource Engineering & Beijing Laboratory of Biomedical Materials & Beijing Advanced Innovation Center for Soft Matter Science and Engineering, Beijing University of Chemical Technology, Beijing, 100029, China.

Table of Content

Materials & Instruments

S1. MSA experiments and assembly of control groups without flexible spacing coatings

S2. Fabrication of the flexible spacing coating and its properties

S3. In situ measurement of the interactive forces

S4. MSA of heterogeneous structures with increased building block number/size

S5. Surface chemistry of building blocks in Figure 2

S6. Fabrication of magnetic-responsive PDMS, PET and Ti building blocks

S7. 3D profile of the 3D ordered structures

Video S1. Stepwise demonstration of the MSA-AM method to construct a 3D ordered structure.

References

Materials & Instruments

The following chemicals were used as supplied: cubic PU, PE, PP, ABS, PS with an edge length of 2~3 mm from Shenzhen Sanze Hardware Plastic Products Co., Ltd., cubic quartz glass from Boyang Quartz Limited Company, cubic Al from Boyang Quartz and Aoshuo Metal Limited Company respectively, poly(ethyleneimine) (PEI, branched, MW=1800) and poly(acrylic acid) (PAA, MW=240000, aq, 25 wt%) from Alfa Aesar, poly-(diallyldimethylammonium chloride) (PDDA, MW=250000~300000, aq, 20 wt%) from Sigma-Aldrich, poly(sodium-p-styrenesulfonate) (PSS, MW=70 000) from Acros Organics, polydimethylsiloxane (PDMS) (Sylgand 184) from Dow Corning, polyethylene terephthalate (PET, thickness: 130 μm) from Deyuan Plastic Products Co., Ltd., Ti sheet (thickness: 50 μm) from Baosheng Hardware Co. Ltd., carboxyl-functionalized Fe_3O_4 magnetic nanoparticles with an average diameter of 240 nm from Shanghai So-Fe Biomedicine Co., Ltd. PAA-CD and PAA-Azo were synthesized as following a previous report[1].

The glass transition temperature of polyelectrolyte multilayers was measured with a differential scanning calorimetry (DSC) method (Mettler-Toledo DCS1). The elastic modulus of used polymer materials was characterized on a universal test machine with tension testing mode. The modulus values of Al and Glass were taken from handbook. Elastic modulus of polyelectrolyte multilayers was measured within deionized water and calculated from force-distance curves obtained from an atomic force microscope (JPK Nanowizard 4, Bruker). Optical photos were taken with a Nikon D5000 camera. Force measurements were performed on a Dynamic Contact Angle Measuring Device and Tensionmeter (DCAT21, Dataphysics). Scanning electron microscopy Zeiss SUPRA55 was used to characterize the morphology of heterogeneous 3D structures at 20 kV, and energy dispersive spectrometer for elemental mapping was conducted with INCA Energy 350 from Oxford Instruments. The 3D profiled images were obtained on a 3D microscope contour GT-X (Bruker).

S1. MSA experiments and assembly of control groups without flexible spacing coatings

MSA experiments were conducted by placing building blocks modified with interactive groups in a container with about 15 mL deionized water and shaken on a rotating shaker at a speed of 180 r/min for 5 min (**Scheme S1**). For every result, at least five independent assembly experiments were repeated. We used cubic building blocks of diverse materials with different densities including: PU (1.115 ± 0.010 g/cm^3), PE (0.825 ± 0.065 g/cm^3), PP (0.856 ± 0.025 g/cm^3), ABS (1.025 ± 0.005 g/cm^3), PS (1.008 ± 0.004 g/cm^3), Al (2.535 ± 0.052 g/cm^3), and glass (2.017 ± 0.099 g/cm^3). The water amount in the container is quite low to just immerse the building blocks. Under shaking conditions, building blocks with different density mainly collide and interct at the bottom of the container, which makes co-assembly possible.

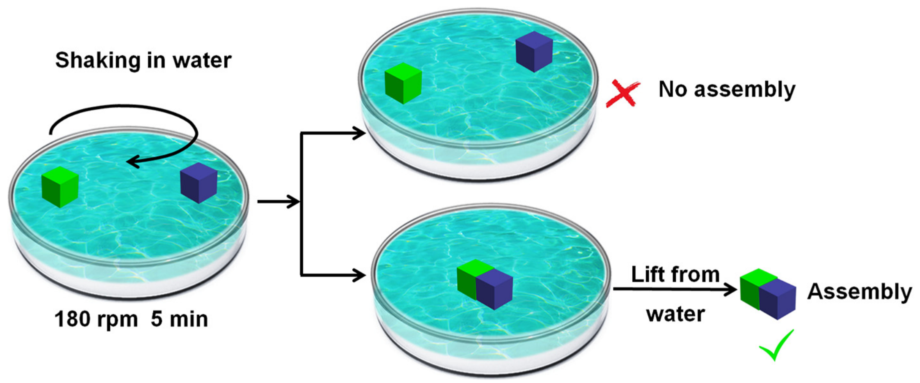

Scheme S1. Schematic illustration of MSA experiments.

When the building blocks were only modified with (PDDA/PAA-CD)$_5$ and (PDDA/PAA-Azo)$_5$ multilayers without the flexible spacing coating, they could not assemble (**Figure S1**).

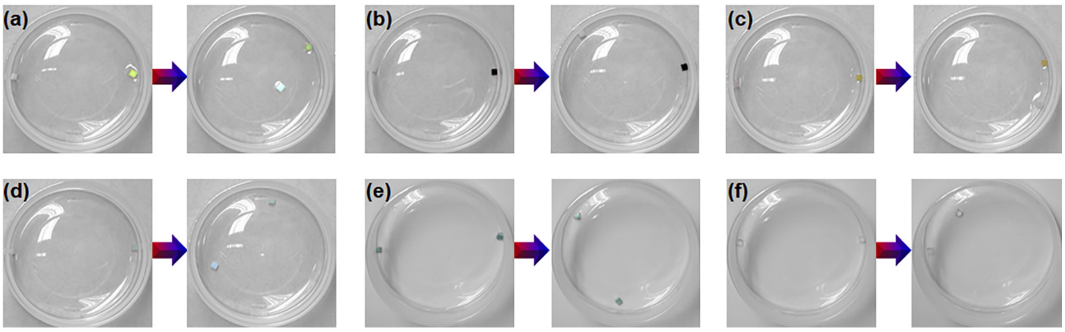

Figure S1. Photos of (a) PU, (b)PE, (c) PP, (d)PS, (e)Al, (f)glass pairs only modified with (PDDA/PAA-CD)$_5$ and (PDDA/PAA-Azo)$_5$ multilayers before and after shaking in water for 5 min at 180 rpm. None are assembled.

S2. Fabrication of the flexible spacing coating and its properties

Fabrication. The flexible spacing coating and supramolecular groups were modified onto building blocks of cubic PU, PE, PP, ABS, PS, Al and quartz glass via a facile layer-by-layer (LbL) method (**Figure 1c**). Taking quartz glass building blocks as an example, firstly, the quartz glass cubes were cleaned with a piranha solution for 30 min and washed with deionized water, followed by immersion in a PEI solution (aq, 1 mg/mL) overnight. Building blocks of other materials were washed with ethanol and deionized water, followed by plasma treatment for 2 min and immersion in a PEI (aq, 1 mg/mL) solution overnight. Subsequently, the building blocks were alternately immersed in PAA (aq, 1 mg/mL) and PEI (aq, 1 mg/mL) solutions for 2 min, between each of which they were rinsed with copious water. The alternate cycles were repeated until designated number (*n*) of multilayers, noted as $(PEI/PAA)_n$. Afterwards, the building blocks were LbL assembled in PDDA (aq, 1 mg/mL) and PSS (aq, 1 mg/mL) for 5 min each, leading to multilayers of $(PDDA/PSS)_n$. Finally, via LbL in PDDA (aq, 1 mg/mL) and PAA-CD (aq, 1mg/mL) or PAA-Azo (aq, 1mg/mL) for 5 min each, the building blocks were modified with a composite multilayer of $(PEI/PAA)_{20}$-$(PDDA/PSS)_{20}$-$(PDDA/PAA\text{-}CD)_5$ or $(PEI/PAA)_{20}$-$(PDDA/PSS)_{20}$-$(PDDA/PAA\text{-}Azo)_5$.

Glass transition temperature (T_g). We obtained the T_g of the composite multilayers of $(PEI/PAA)_{20}$ and $(PEI/PAA)_{20}$-$(PDDA/PSS)_{20}$ with DSC measurements. The samples for DSC tests were prepared following the reported procedure[2]. The films were deposited onto polytetrafluoroethylene substrates, which allows for the easy peeling-off and collection of the films in water. The as-collected films after removing apparent water were used for DSC tests by referring to the reported test methods[3,4]. About 5 mg of collected films in a hydrated state were sealed in the pan of the DSC instrument and equilibrated at 0 °C for 5 min, and then ramped from 0 to 115 °C at a rate of 2 °C/min; the thermal cycle was repeated two times and the results of the second heating scans were taken as shown in **Figure S2**. The T_gs of both $(PEI/PAA)_{20}$ and $(PEI/PAA)_{20}$-$(PDDA/PSS)_{20}$ multilayers are about 10 °C, which indicates a

high motility of these polyelectrolytes at room temperature and thus favors for providing a highly compliant surface for efficient multivalency of the outmost supramolecular groups.

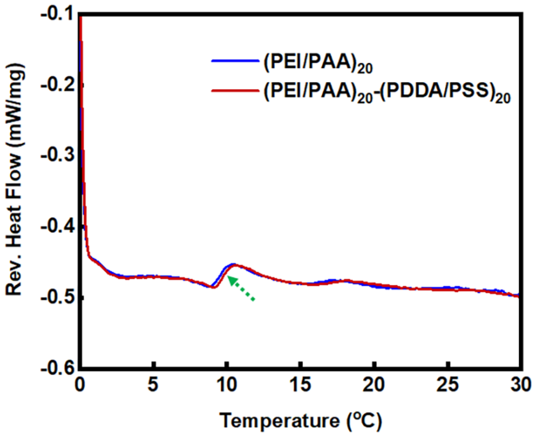

Figure S2. DSC curves of hydrated $(PEI/PAA)_{20}$ (blue line) and $(PEI/PAA)_{20}$-$(PDDA/PSS)_{20}$ (red line) multilayers. The arrow indicates the transition at 10 °C.

Young's modulus. We calculated the Young's modulus of the flexible spacing coating from AFM force-distance curves obtained from tests in deionized water when varying the deposition cycles of PEI/PAA. Quartz substrates modified with $(PEI/PAA)_x$-$(PDDA/PSS)_{20}$-$(PDDA/PAA\text{-}Azo)_5$ multilayers (x=0, 5, 10, 15, and 20) were tested and the Young's moduli are 107.4 ± 51.3, 62.7 ± 27.2, 24.9 ± 19.4, 17.6 ± 7.5, and 17.5 ± 12.9 MPa, respectively. The Young's modulus dropped from about 107 MPa without the PEI/PAA multilayer to about 17.5 MPa with 20 bilayer of PEI/PAA, indicating increased surface compliance.

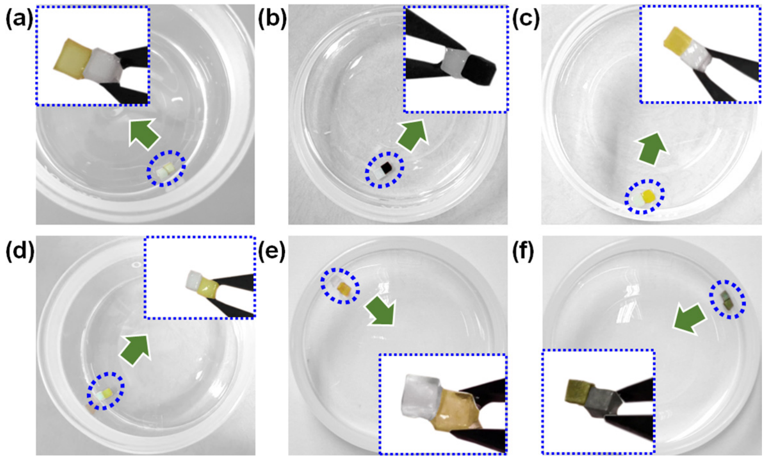

Figure S3. Photos of (a) PU, (b)PE, (c) PP, (d)PS, (e) Al, (f) quartz glass building blocks after shaking in water for 5 min at 180 rpm. Each dimer is assembled from an interactive pair of

building blocks modified with $(PEI/PAA)_{20}$-$(PDDA/PSS)_{20}$-(PDDA/PAA-CD or $Azo)_5$ and $(PEI/PAA)_{20}$-$(PDDA/PSS)_{20}$-(PDDA/PAA-CD or $Azo)_5$ multilayers, respectively.

Effects on facilitating MSA. After pre-modifying the flexible spacing coatings of $(PEI/PAA)_{20}$-$(PDDA/PSS)_{20}$ beneath the supramolecular multilayers of $(PDDA/PAA\text{-}CD)_5$ or $(PDDA/PAA\text{-}Azo)_5$, building blocks assembled into dimers and could be lifted out of water immediately after assembly (**Figure S3**).

To exclude the possibility of self-stickiness, control MSA experiments of identical building blocks with the same groups were conducted. No assembly was observed (**Figure S4**).

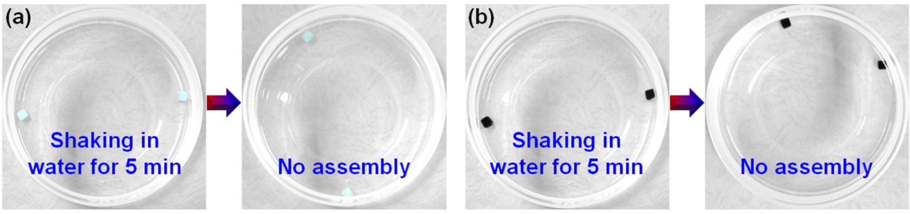

Figure S4. Photos of ABS building blocks modified with (a) $(PEI/PAA)_{20}$-$(PDDA/PSS)_{20}$-$(PDDA/PAA\text{-}CD)_5$ and (b) $(PEI/PAA)_{20}$-$(PDDA/PSS)_{20}$-$(PDDA/PAA\text{-}Azo)_5$ before and after shaking in water for 5 min at 180 rpm.

S3. In situ measurement of the interactive forces

The interactive forces between building blocks were in situ measured in water based on a controlled contact-detachment process between interactive pairs (**Figure S5a**) with a DCAT apparatus (**Figure S5b**). As schematically illustrated in **Figure S5a**, the measurement consists of a force sensor at the top, a cell containing water and building blocks for measurements, and a motor-driven stage with controlled moving velocity of 0.5 mm/s. The cube and the sheet were an interactive pair modified with designated multilayers to ensure a constant contacting area in each contact-detachment test. To increase the density of the cube for immersion in water for stable tests, extra PDMS cubes loaded with metal are added (**Figure S5b**). The whole test process was conducted within water. In the beginning, the cube and sheet building blocks were separate and the force sensor was adjusted to a zero state to indicate a balanced force exerted

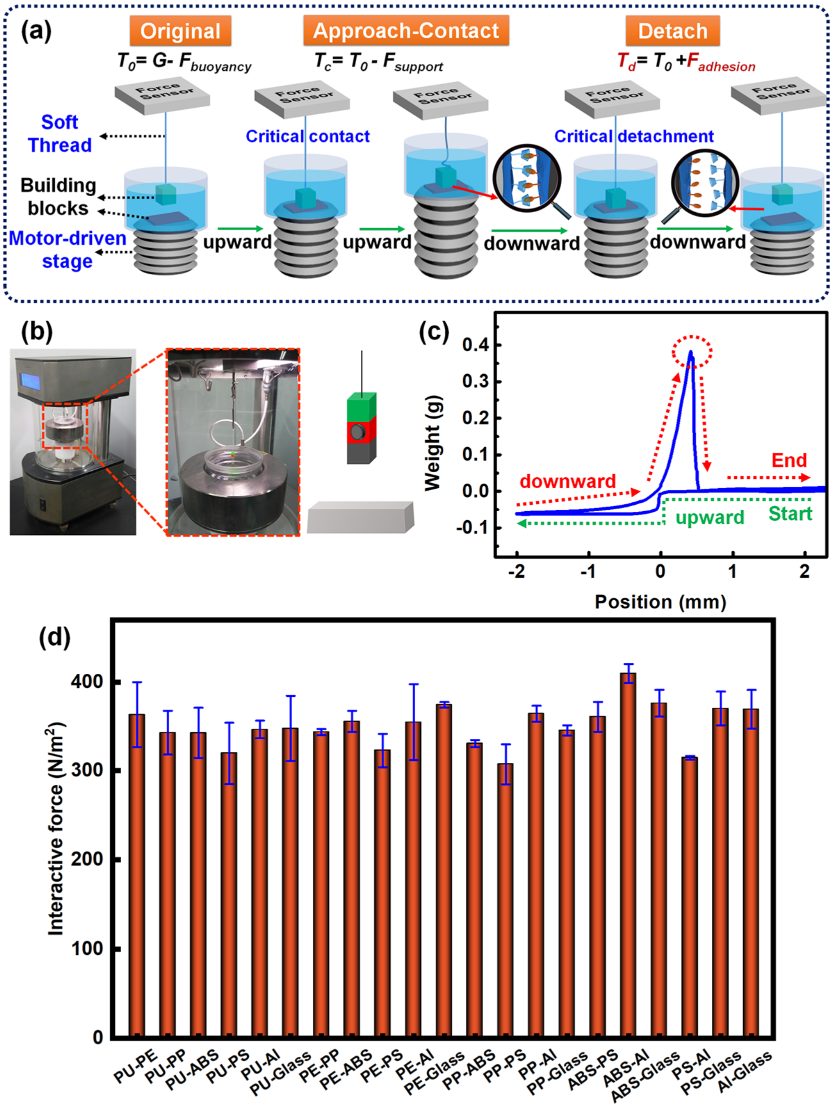

Figure S5. (a) Schematic illustration of the principle of the in situ force measurement. (b) Photos of the force measurement apparatus and schematic illustration of building blocks in force measurement. (c) A typical force curve of interactive host/guest building blocks in one test. (d) Interactive force values between heterogenous materials. 'PU-PE' means a PU building block modified with a $(PEI/PAA)_{20}$-$(PDDA/PSS)_{20}$-$(PDDA/PAA\text{-}CD)_5$ multilayer interacted with a PE building block modified with a $(PEI/PAA)_{20}$-$(PDDA/PSS)_{20}$-$(PDDA/PAA\text{-}Azo)_5$ multilayer.

on the cube. The tension of the soft thread reflects the force changes on the suspended green cubic building block: the tension T_0 is balanced with gravity and buoyancy, which could be taken as a reference; subsequently, the motor drives the container of the other red building block upward to approach and contact the green cube, during which the cube is totally supported by the stage and the thread lost its tension and thus the two building blocks could have sufficient molecular recognition; afterwards, the motor drives downward to result in the detachment and

sepration at a critical point that reveals the adhesion strength ($F_{adhesion}$) between the two building blocks; finally these building blocks are totally separated and back to the original state. With reference to T_0 and the recorded T_d values, the adhesion strength ($F_{adhesion}$) could be obtained from a typical force curve in one test (**Figure S5c**). We normalized this value relative to interacted surface area and obtained the results of binding between distinctive materials in **Figure S5d.** By modifying the building blocks with the flexible spacing coating and supramolecular interactive groups, most interactive pairs of heterogeneous materials (e. g. PU-PE, PU-PP, PU-ABS etc.) display an adhesion strength of 300~400 N/m^2, which is at a similar level to that of identical materials.

The bilayer number of the PEI/PAA multilayer matters in the binding strength of the assembled interface. We have measured the in situ interactive forces between ABS building blocks modified with the composite multilayers with different bilayer number of the PEI/PAA multilayer as follows: (PDDA/PSS)$_{20}$-(PDDA/PAA-CD or Azo)$_5$; **(PEI/PAA)$_5$**-(PDDA/PSS)$_{20}$-(PDDA/PAA-CD or Azo)$_5$; **(PEI/PAA)$_{10}$**-(PDDA/PSS)$_{20}$-(PDDA/PAA-CD or Azo)$_5$; **(PEI/PAA)$_{15}$**-(PDDA/PSS)$_{20}$-(PDDA/PAA-CD or Azo)$_5$; **(PEI/PAA)$_{20}$**-(PDDA/PSS)$_{20}$-(PDDA/PAA-CD or Azo)$_5$.

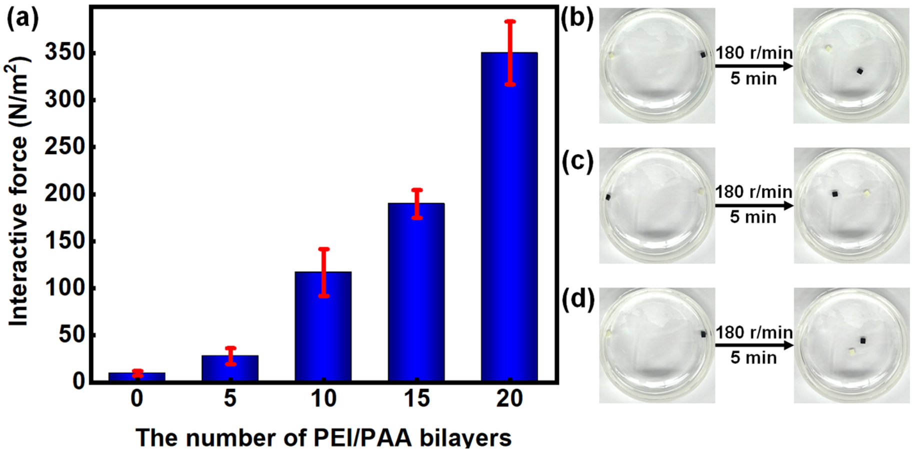

Figure S6. (a) Interactive force between ABS building blocks with varied (PEI/PAA)$_x$ multilayer in the composite films of **(PEI/PAA)$_x$**-(PDDA/PSS)$_{20}$-(PDDA/PAA-CD or Azo)$_5$ (x=0, 5, 10, 15, 20). MSA results of ABS building blocks with (b) **(PEI/PAA)$_5$**-(PDDA/PSS)$_{20}$-(PDDA/PAA-CD or Azo)$_5$, (c) **(PEI/PAA)$_{10}$**-(PDDA/PSS)$_{20}$-(PDDA/PAA-CD or Azo)$_5$, and (d) **(PEI/PAA)$_{15}$**-(PDDA/PSS)$_{20}$-(PDDA/PAA-CD or Azo)$_5$.

From the results summarized in **Figure S6a**, we could observe a force increase along with the increased LbL cycle number of the PEI/PAA multilayer. The interactive force is as low as about 180 N/m^2 when the (PEI/PAA)$_{15}$ case, which is almost a half of the force value in the case of (PEI/PAA)$_{20}$ we used in this work. Correspondingly, we conducted qualitative MSA experiments when the PEI/PAA multilayer has a cycle number of 5, 10, and 15 (**Figure S6b-d**). No assembly was observed. We followed the principle of realizing MSA with minimum deposition cycles of multilayers and determined the final condition as (PEI/PAA)$_{20}$-(PDDA/PSS)$_{20}$.

S4. MSA of heterogeneous structures with increased building block number/size

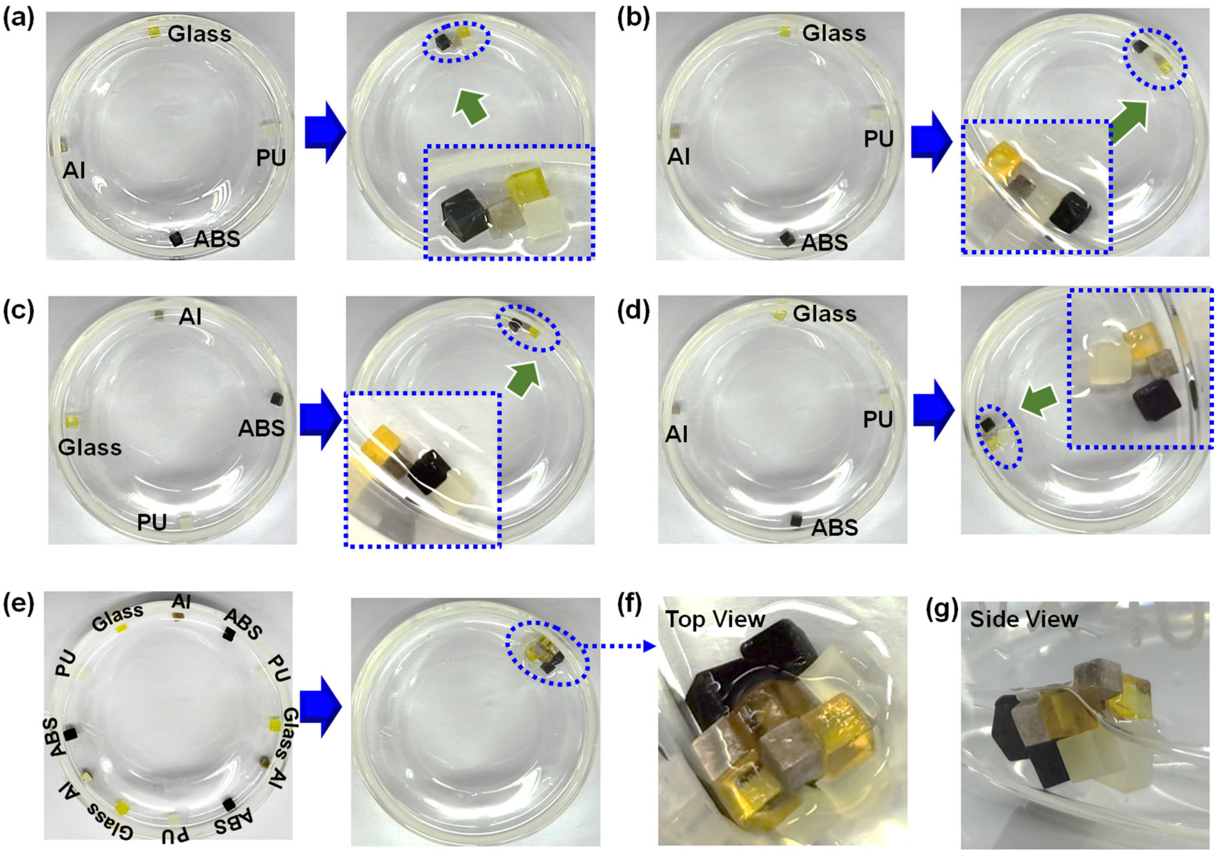

Figure S7. (a-d) Four independent parallel assembly experiments of four distinctive building blocks of Al, ABS, Glass and PU before and after shaking in water for 5 min at 180 r/min. (e) MSA of 3 Al, 3 ABS, 3 Glass and 3 PU building blocks. Magnified pictures of (f) top view and (g) side view of the assembled aggregates. Note that among all the above building blocks, PU and Al were modified with a (PEI/PAA)$_{20}$-(PDDA/PSS)$_{20}$-(PDDA/PAA-CD)$_5$ multilayer while ABS and Glass were modified with a (PEI/PAA)$_{20}$-(PDDA/PSS)$_{20}$-(PDDA/PAA-Azo)$_5$ multilayer.

The geometry of MSA structures assembled from four building blocks (Al, Glass, PU, ABS) is diverse including linear or L-shaped morphology (**Figure S7a-d**). It is hard to control the geometry of the assembled structures because of the complex fluidic dynamics under shaking conditions. With the increased building block number, their MSA normally leads to an aggregate, in which the building blocks are linked via a host/guest connection. As shown in **Figure S7e-g**, the PU and Al building blocks were modified with a $(PEI/PAA)_{20}$-$(PDDA/PSS)_{20}$-$(PDDA/PAA\text{-}CD)_5$ multilayer while the ABS and Glass building blocks were modified with a $(PEI/PAA)_{20}$-$(PDDA/PSS)_{20}$-$(PDDA/PAA\text{-}Azo)_5$ multilayer.

We have demonstrated the assembly of larger building blocks with a dimension of centimeter (**Figure S8**). Four distinct materials were used as building blocks with different geometries: PP (Yang shape of a Taiji), ABS (Yin shape), PS and ABS (white and black disks with a dimeter of 5 mm). The Yin-shaped ABS and the black ABS disk were modified with $(PEI/PAA)_{20}$-$(PDDA\text{-}PSS)_{20}$-$(PDDA/PAA\text{-}Azo)_5$; meanwhile the Yang-shaped PP and the white PS disk were modified with $(PEI/PAA)_{20}$-$(PDDA\text{-}PSS)_{20}$-$(PDDA/PAA\text{-}CD)_5$. After manually connecting them together, we could lift the whole Taiji pattern immediately after their assembly. This result indicates that this methodology could be upscaled to assemble larger structures.

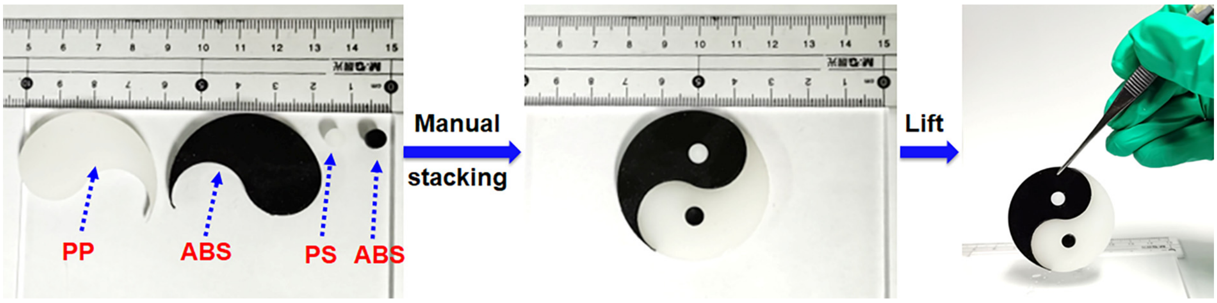

Figure S8. Manually assisted MSA of distinctive building blocks of PP, ABS and PS into a Taiji pattern.

S5. Surface chemistry of building blocks in Figure 2

Table S1. Surface Chemistry of Each Building Block in Figure 2

Structure	Material	Multilayer Components
Dimer (Figure 2a)	Al	$(PEI/PAA)_{20}$-$(PDDA/PSS)_{20}$-$(PDDA/PAA\text{-}CD)_5$
	Glass	$(PEI/PAA)_{20}$-$(PDDA/PSS)_{20}$-$(PDDA/PAA\text{-}Azo)_5$
Dimer (Figure 2b)	PU	$(PEI/PAA)_{20}$-$(PDDA/PSS)_{20}$-$(PDDA/PAA\text{-}CD)_5$
	ABS	$(PEI/PAA)_{20}$-$(PDDA/PSS)_{20}$-$(PDDA/PAA\text{-}Azo)_5$
Dimer (Figure 2c)	PU	$(PEI/PAA)_{20}$-$(PDDA/PSS)_{20}$-$(PDDA/PAA\text{-}CD)_5$
	Glass	$(PEI/PAA)_{20}$-$(PDDA/PSS)_{20}$-$(PDDA/PAA\text{-}Azo)_5$
Dimer (Figure 2d)	PU	$(PEI/PAA)_{20}$-$(PDDA/PSS)_{20}$-$(PDDA/PAA\text{-}CD)_5$
	Al	$(PEI/PAA)_{20}$-$(PDDA/PSS)_{20}$-$(PDDA/PAA\text{-}Azo)_5$
Trimer (Figure 2e)	PU	$(PEI/PAA)_{20}$-$(PDDA/PSS)_{20}$-$(PDDA/PAA\text{-}CD)_5$
	Al	$(PEI/PAA)_{20}$-$(PDDA/PSS)_{20}$-$(PDDA/PAA\text{-}Azo)_5$
	Glass	$(PEI/PAA)_{20}$-$(PDDA/PSS)_{20}$-$(PDDA/PAA\text{-}Azo)_5$
Tetramer (Figure 2f)	Al	$(PEI/PAA)_{20}$-$(PDDA/PSS)_{20}$-$(PDDA/PAA\text{-}CD)_5$
	Glass	$(PEI/PAA)_{20}$-$(PDDA/PSS)_{20}$-$(PDDA/PAA\text{-}Azo)_5$
	PU	$(PEI/PAA)_{20}$-$(PDDA/PSS)_{20}$-$(PDDA/PAA\text{-}CD)_5$
	ABS	$(PEI/PAA)_{20}$-$(PDDA/PSS)_{20}$-$(PDDA/PAA\text{-}Azo)_5$

S6. Fabrication of magnetic-responsive PDMS, PET and Ti building blocks

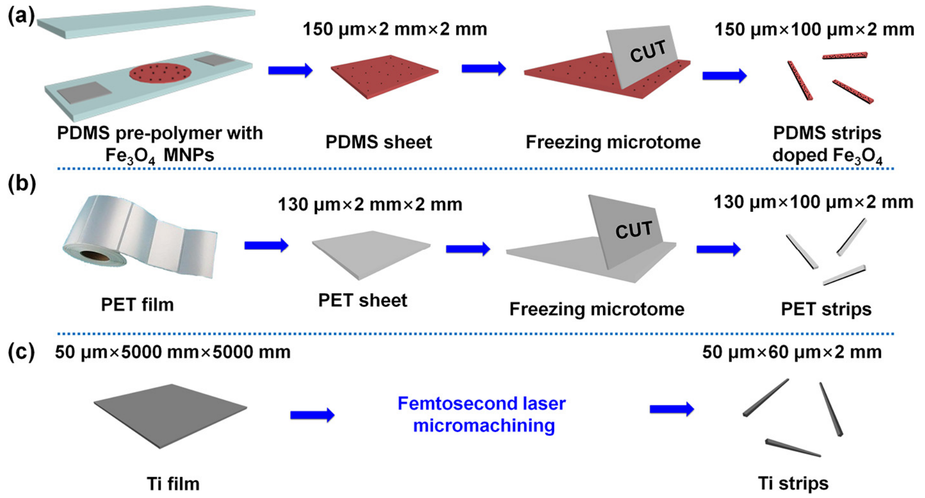

Figure S9. Schematic illustration of the fabrication processes of strip-like building blocks: (a) PDMS, (b) PET, (c) Ti.

PDMS, PET and Ti were prepared into a dimension of 150 μm × 100 μm × 2 mm, 130 μm × 100 μm × 2 mm and 50 μm × 60 μm × 2 mm, correspondingly, following the procedures shown in **Figure S9**. To be specific, degassed mixture of PDMS pre-polymer and its curing agent (the weight ratio is 10:1) was poured between two hydrophobic glass slides sandwiched by cover glasses with a thickness of 150 μm, followed by curing at 70 °C for 1 h. PET and Ti films with

a thickness of 130 μm and 50 μm are commercially available. Thin films of PDMS and PET were pre-cut into sheets with a size of 2 mm × 2 mm with blades and further cut into strips with a width of 100 μm with a freezing microtome. The Ti film was machined via femtosecond laser micromachining techniques.

For the fabrication of magnetic-responsive PDMS building blocks, dry particles of Fe_3O_4 MNPs were incorporated via directly mixing in the prepolymer mixture before curing. For PET and Ti, we applied the LbL method to deposit multilayers of PDDA and carboxyl-functionalized Fe_3O_4 magnetic nanoparticles (**Figure S10**). The strip building blocks were cleaned by ultrasonication in ethanol for 10 min, rinsed with deionized water and dried in nitrogen flow, followed by plasma treatment for 5 min and immersion in PDDA (aq, 1 mg/mL) overnight to introduce a layer of positive charges. Then the building blocks are washed with deionized water and alternately immersed in PDDA (aq, 1 mg/mL) and Fe_3O_4 nanoparticles (aq, 0.5 mg/mL) for 5 min each for cycles, between which deionized water was used for rinses. Finally, PET and Ti building blocks were modified with a (PDDA/Fe_3O_4 MNPs)$_5$ multilayer.

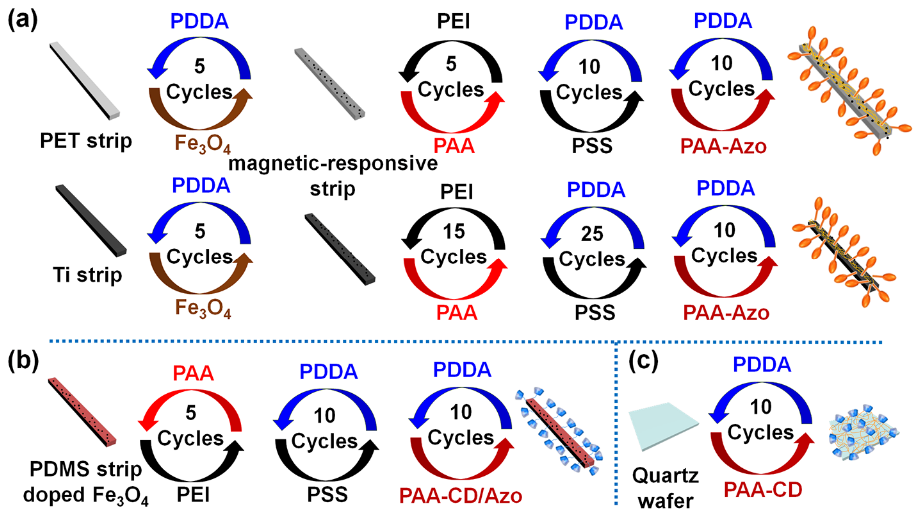

Figure S10. (a) Schematic illustration of fabrication of magnetic-responsive PET and Ti building blocks and further modification of flexible spacing coatings and Azo groups; surface modification of (b) PDMS building blocks with flexible spacing coatings and CD groups, and (c) quartz substrate with PDDA/PAA-CD multilayers.

Subsequently, the above magnetic-responsive building blocks were further modified with flexible spacing coatings and supramolecular groups by LbL: PDMS with a $(PEI/PAA)_5$-$(PDDA/PSS)_{10}$-(PDDA/PAA-CD or $Azo)_{10}$ multilayer, PET with a $(PEI/PAA)_5$-$(PDDA/PSS)_{10}$-$(PDDA/PAA\text{-}Azo)_{10}$ multilayer and Ti with a $(PEI/PAA)_{15}$-$(PDDA/PSS)_{25}$-$(PDDA/PAA\text{-}Azo)_{10}$ multilayer. Besides, quartz substrates (3 cm × 1 cm × 1 mm) were modified with a $(PDDA/PAA\text{-}CD)_{10}$ multilayer to support the 3D ordered structures.

The stepwise LbL assembly of Fe_3O_4 MNPs was characterized by UV-vis absorption spectroscopy by tracing a wide absorption at around 480 nm (**Figure S11**). The inset shows a linear correlation between the absorbance of Fe_3O_4 MNPs at 480 nm and number of PDDA/Fe_3O_4 MNPs bilayers.

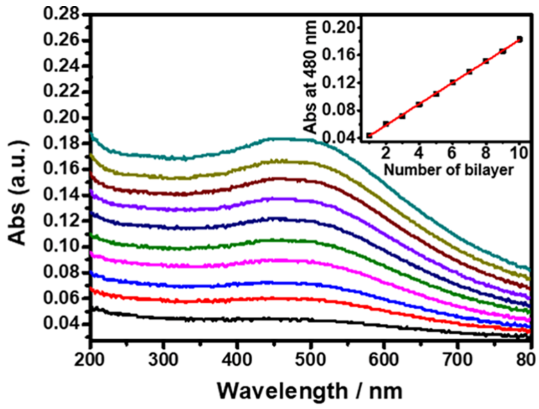

Figure S11. Stepwise UV−visible spectra of the PAA/Fe_3O_4 MNPs multilayer. The inset shows a linear correlation between the absorbance at 480 nm and the number of deposited bilayers.

S7. 3D profile of the 3D ordered structures

The assembly precision of the 3D structure fabricated by the MSA-AM method is characterized by a 3D profiler (**Figure S12**) with calculations of location and angular deviation of the building blocks. For example, for the first layer with four parallel strip building blocks, the center-to-center distance of two adjacent building blocks was measured, leading to an averaged location deviation of about 3.33 μm. We took the first strip building block as reference, and measured the angular deviation of the subsequent three building blocks, leading to an averaged deviation value of about 0.87°.

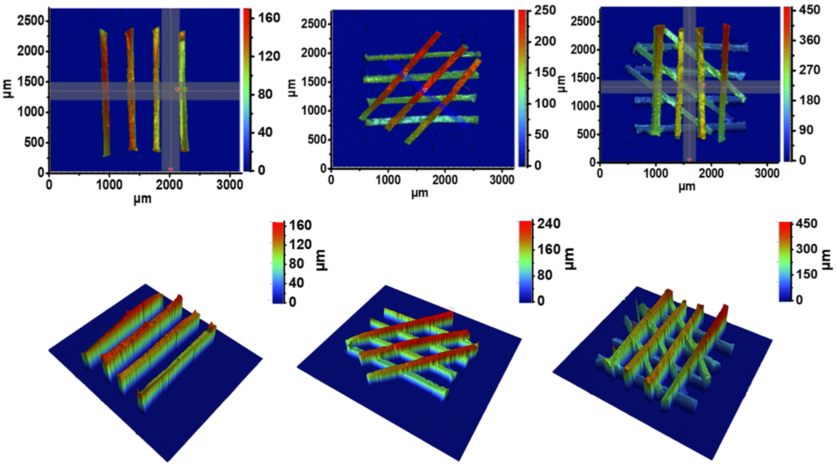

Figure S12. 3D profile images of heterogeneous 3D structures with alignment of one, two and three layers.

References

[1] M. J. Cheng, F. Shi, J. S. Li, Z. F. Lin, C. Jiang, M. Xiao, L. Q. Zhang, W. T. Yang, T. Nishi, *Adv. Mater.* **2014**, *26*, 3009.

[2] J. L. Lutkenhaus, K. D. Hrabak, K. McEnnis, P. T. Hammond, *J. Am. Chem. Soc.* **2005**, *127*, 17228.

[3] D. K. Reid, A. Summers, J. O'Neal, A. V. Kavarthapu, J. L. Lutkenhaus, *Macromolecules* **2016**, *49*, 5921.

[4] Y. Zhang, P. Batys, J. T. O'Neal, F. Li, M. Sammalkorpi, J. L. Lutkenhaus, *ACS Cent. Sci.* **2018**, *4*, 638.
